# Mortality risk in patients with myasthenia gravis

**DOI:** 10.3389/fneur.2025.1586031

**Published:** 2025-05-14

**Authors:** Mohamed Khateb, Shahar Shelly

**Affiliations:** ^1^Department of Neurology, Rambam Medical Center, Haifa, Israel; ^2^Department of Neurology, University Health Network (UHN), University of Toronto, Toronto, ON, Canada; ^3^Neuroimmunology Laboratory, Ruth and Bruce Rappaport Faculty of Medicine, Technion – Israel Institute of Technology, Haifa, Israel; ^4^Department of Neurology, Mayo Clinic, Rochester, MN, United States

**Keywords:** myasthenia gravis, mortality, survival, myasthenic crisis, epidemiology, thymectomy

## Abstract

**Introduction:**

Although some reports link Myasthenia Gravis to higher mortality, the evidence remains contradictory and unclear. Real-life data is limited primarily due to challenges in selecting control groups and mitigating bias. Additionally, a revised mortality assessment should be conducted due to recent advancements in Myasthenia Gravis treatments over the past decade, including new biological therapies and the impact of the COVID-19 pandemic from 2020 to 2023.

**Methods:**

We conducted a retrospective analysis of all patients diagnosed with Myasthenia Gravis at our tertiary center between 2000 and 2023, extracting mortality and clinical features compared to two age- and sex-matched control groups of neurological or rheumatologic patients.

**Results:**

We identified 436 Myasthenic patients and 2,616 controls (1308 in each control group). Myasthenia Gravis mortality was 14% at 5 years (61/422) and 21% at 10 years (87/422). Mortality was significantly higher than control groups (*p* < 0.001). Intubations during myasthenic crisis were linked to higher mortality (*p* = 0.002). Bulbar weakness at presentation showed higher mortality but did not reach clinical significance. We compared the mean age at death in MG patients to national life expectancy benchmarks using a one-sample Z-test, revealing significantly younger age at death in both males (78.3 vs. 81.6 years, *p* = 0.009) and females (76.5 vs. 85.2 years, *p* < 0.00001). Patients with normal thymic pathology showed better outcomes and lower mortality after thymic removal (*p* < 0.0001). The primary cause of death was linked to infections, significantly correlated with chronic steroid use.

**Discussion:**

In conclusion, patients with Myasthenia Gravis had higher mortality rates. Thymic removal reduced mortality, while intubation is associated with increased mortality risk.

## Introduction

Mortality data for myasthenia gravis (MG) is limited, and assessing it remains challenging due to the disorder’s clinical heterogeneity. Death may result from myasthenic crises, which affect approximately 15%–20% of patients over their lifetime, along with the contribution of unrelated vascular risk factors (1–3). Few studies have suggested that the overall mortality rate is not significantly different from the general population with most deaths occurring in those with severe forms of the disorder during the first 2 years (4, 5) and were more common in males (5). Older studies from the end of the 20th century showed higher mortality to that of the general population in a Danish cohort of 290 patients (6). With the advancement of treatments and medical care, mortality was expected to decline. One large US nationwide study with more than 5,000 patients showed that the overall in-hospital mortality rate was 2.2% with older age and respiratory failure as risk factors. This was eventually concluded as a low mortality rate (4). Similarly, a recent 2020 study from four Swedish National Board of Health and Welfare registers showed similar mortality rates to the general population (7). However, there were contradicting studies that proposed increased mortality. A recent study of more than 4,000 patients in China demonstrated that MG-related mortality was high, most dramatically at ages of 10–19 and over 70 years (8). A similar trend of results was shown by studies of Hansen et al. (9), further supporting the association of MG with increased mortality.

Previous studies have demonstrated several associations related to mortality in MG. These include the occurrence of high severity and long duration of MG, myasthenic crises, respiratory failure, and other severe complications arising from delayed or inadequate treatment (2, 4). Up to 85% of MG patients can have thymic abnormalities (10), and thymic removal even in the absence of abnormality is routinely recommended in seropositive acetylcholine receptor (AChR) antibodies early-onset patients (11). A recent study reviewing a large cohort of adult patients who underwent thymectomy showed thymic removal was associated with excess mortality (12). This study suggested thymus is essential for immune competence and overall health. The researchers analyzed 1,420 patients, most of whom did not have MG, who underwent thymectomy. Five years post-surgery, the thymectomy group showed higher all-cause mortality (8.1% vs. 2.8%) and an increased risk of cancer (7.4% vs. 3.7%). These findings raise the important question of whether thymectomy may act as a trigger for higher mortality, particularly in MG patients, and whether this increased risk warrants further investigation. Other studies showed an association with older age, respiratory failure, the presence of thymoma, and high titers of AChR antibodies (2, 4, 6). Considering the advances in our understanding of the care and treatment of patients with MG, one might expect that the mortality rates will show a declining trend over time. However, multiple investigations into MG mortality, including recently published studies, as reported by Chen Zhang in 2023 and Vissing in 2024, have failed to substantiate this expectation (8, 9, 13, 14).

This study aims to estimate age and sex-adjusted mortality rates for MG accurately. It will compare these mortality rates with those of age and sex-matched neurological and rheumatological control cohorts and explore potential associations with mortality.

## Methods

### Study design, ethical considerations, and study cohort

The Rambam Medical Center Helsinki Committee approved this study.

We included patients with a confirmed diagnosis of MG, at a tertiary medical facility located in Haifa, northern Israel, spanning the timeframe from January 1, 2000, to May 31, 2023. The gold standard for an MG diagnosis was the combination of both a clinical impression of MG and a supportive electrodiagnostic (EDX) testing or serology [Ach receptor verified by cell-based-arrays/CBAs or muscle-specific tyrosine-kinase (MuSK)]. Notably, we also requested the absence of any alternative diagnosis explaining the clinical presentation, the laboratory, or the electrodiagnostic findings. Two-Hertz motor repetitive nerve stimulation (RNS) with a train of 4 stimuli was deemed confirmatory when a decrement of >10% was seen in the compound muscle action potential at baseline or up to 3 min post 1 min exercise in 2 or more motor nerves (15). Stimulatory single-fiber EMG (SFEMG) was also performed in suspected patients. SFEMG positivity was determined utilizing quality and cutoff guidelines previously published (16, 17). A double seronegative serologic status was not considered an exclusion criterion as part of myasthenic patients are known to be seronegative.

### Data compilation

Pertinent demographic and clinical particulars were carefully garnered from the medical dossiers of the eligible subjects. Details concerning mortality were ascertained via cross-referencing with our hospital-based critical vital statistics archives. A comprehensive and thorough evaluation of each case was undertaken, with experienced neurologists (M.KH, and S.S) for the chart review process, ensuring accuracy. Notably, relying on the existing national vital event tracking system, all deaths of those patients were recorded, even deaths outside our institute.

### Control matching

Control group analysis was performed similarly to our recently published article (18), using facilitated employing the MDClone platform (MDClone Ltd., Beer Sheva, Israel). The MDClone software, seamlessly integrated into our electronic medical records (EMR) system, provides a user-friendly, self-serviced data analytics environment. This empowers the formulation of intricate search queries and streamlines access to the complete repository of retrospective hospital data. For this study, two distinct control clusters were examined, with a specific focus on age and sex harmonization (18). These groups encompassed patients without diagnosis of myasthenia gravis, referred to either the neurological or rheumatological departments within the same tertiary healthcare facility. Data extraction process was tailored to align with age and sex parameters while ensuring the exclusion of myasthenic patients. The resultant dataset was comprised of 1,308 individuals from each of the control groups. Specifically, for each myasthenia gravis patient within the cohort, three random patients exhibiting matching age and sex attributes were systematically gathered from each of the control cohorts.

### End points and statistical analysis

We looked at all causes of deaths as our primary endpoint of the study. Descriptive statistics were used to characterize participant demographics, while categorical variables were analyzed using chi-square or Fisher’s exact tests (significance: *p* < 0.05). Multiple linear regression analysis was employed to identify significant predictors of mortality. Group differences with ages were established using Wilcoxon statistics between the means of continuous variables. Survival was analyzed, using Kaplan–Meier and Cox regression analysis following further grouping of our patients into those who died or survived. *p* < 0.05 was regarded as statistically significant. All statistical analyses were performed using R software (version 4.1.2). Kaplan–Meier survival curves were used to estimate the probability of survival over time. Matching between the MG cohort and the control groups was performed according to age and sex. Specifically, the statistical methodology used for the age-and-sex matching was “nearest neighbor matching.”

### Data availability full data access statements

Authors take full responsibility for the data, the analyses and interpretation, and the conduct of the research; they have full access to all the data; and that they have the right to publish all data. Anonymized data not published within this article will be made available by request from any qualified investigator.

## Results

### Patient characteristics, electrodiagnostic and clinical features

During the time of the study, 539 patients with suspected MG were initially identified, of which 103/539 did not meet the inclusion criteria (see the Methods) and thus were excluded at the beginning. Of these, 51 were excluded due to alternative diagnoses better explaining the clinical presentation like diabetic ophthalmopathy, brainstem vascular or demyelinating lesions, myopathy, and more. Fifty-three patients without an alternative diagnosis were excluded due to insufficient clinical, electrodiagnostic and serologic details to confirm the MG diagnosis. We identified 436 patients with MG diagnosis. Median age at symptoms onset was 64 (range: 5–93 years) for males and 54 (range: 1–87 years) for females. MG symptoms at onset were recorded as ocular (59%, 257/436), strictly bulbar (10%, 45/436) or generalized (23%, 99/436). The remaining 8% of patients’ symptoms were not well characterized. Median follow-up time was 3 years and the mean was 5.21 years (range: 1–34 years). Validation of serological status was achieved by re-testing in 51% of cases, out of which, 78% were seropositive (168/222, 76% for AChR and 6/222, 3% for MuSK) and the remaining 22% (48/222) double-seronegative. Electrophysiological testing was available for review in 67% (294/436) of patients who underwent SFEMG or RNS or both (SFEMG 99%, 291/294 or RNS 35%,102/294). RNS was abnormal in 42% (43/102) and SFEMG was abnormal in 89% (259/291). Out of the 291 patients that had SFEMG study, in 128 of them, the study itself was not available for review but only the final interpretation. The mean jitter in the abnormal group was 38.33 μs (range: 18–167 μs) among the positive patients. Chest Computed Tomography (CT) was performed in 96% (420/436) and was abnormal in 31% (136/436) raising a radiological suspicious of thymic mass or hyperplasia. Thymic removal was carried out in 32% (140/436) of patients, and in 9.2% (40/436) the histopathological report confirmed thymoma.

### Mortality from symptoms onset, sex differences, and controls

Death occurred in 31% (133/436) of MG patients, there were no sex differences, with 52% (69/133) males and 48% (64/133) females (*p* = 0.2). MG patients died younger compared with the epidemiological data in Israel both males and females (19). Median death age was 79 years as a group (range: 32–107 years), 83 years for males (range: 32–107 years) and 79 (44–106 years) for females (*p* = 0.42). Average age of death was 78.3 and 76.5 years for MG males and females, respectively, vs. 80.7 and 84.6 in the general population of Israel. We performed a one-sample Z-test comparing the average age at death in MG patients to national life expectancy benchmarks. Among males, the mean age at death was 78.3 years, compared to an expected 81.6 years, yielding a statistically significant difference (Z = −2.61, *p* = 0.009). Among females, the mean age at death was 76.5 years vs. 85.2 years expected, also statistically significant (Z = −6.63, *p* < 0.00001). Note that this comparison is not risk-adjusted, so part of the difference could stem from non-MG-related mortality. This is based on the nation statistical data base: https://www.cbs.gov.il/he/publications/DocLib/2023/1911_life_tables_2017_2021/e_print.pdf.

Five years from symptoms onset, 14% (61\422) of patients died at the median age of 80 years (32–97 years). Ten years from symptoms onset 21% (87\422) of patients died with median age at death of 81 years (32–97 years).

**Figure 1:**
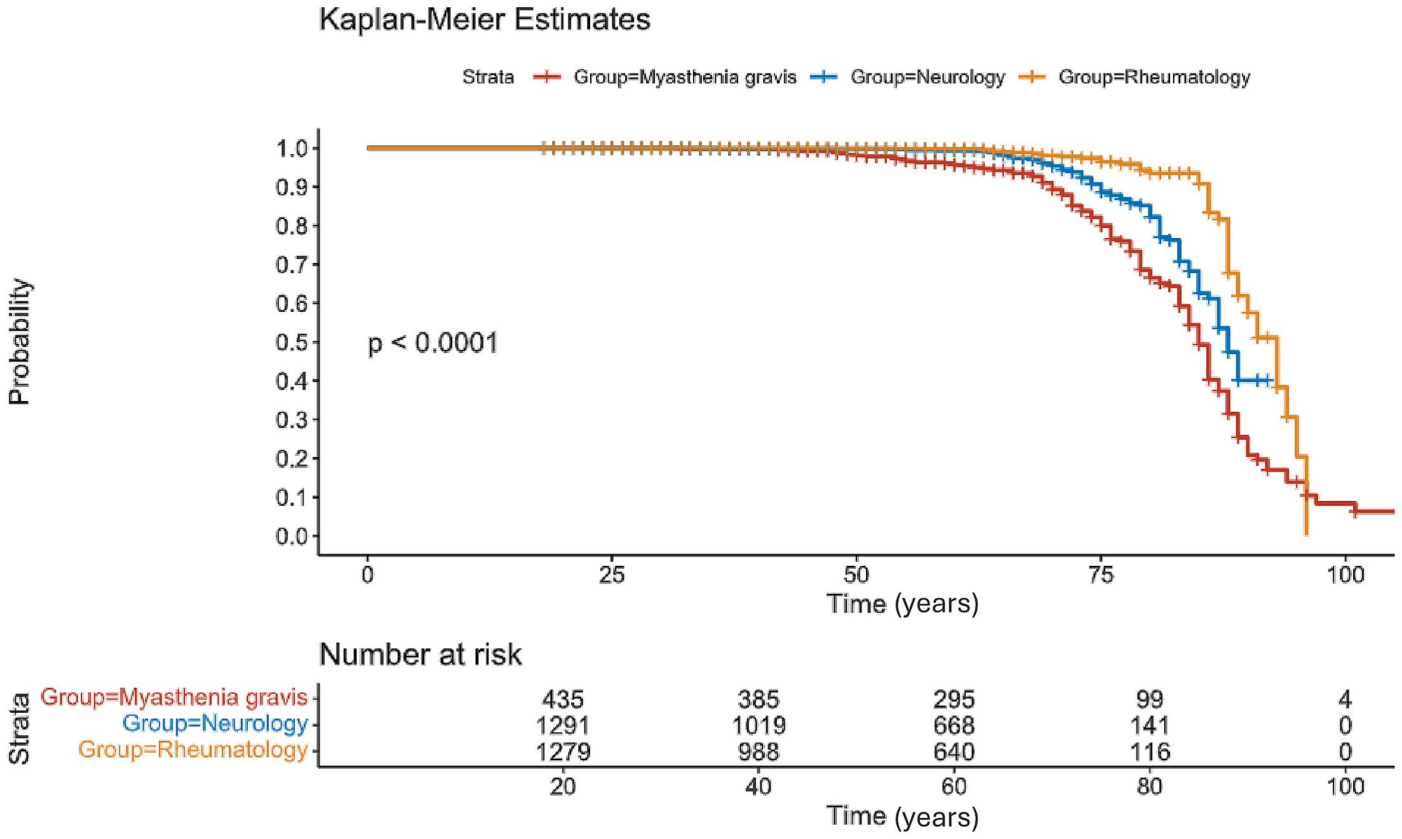
Survival from all cause’s comparison: Kaplan–Meier survival analysis comparing the overall survival between patients with MG (in red) to controls (neurology and rheumatology) matched from the time of diagnosis be age and sex. MG cohort shows the highest mortality. The informative risk table set (bottom) displays the number of patients that were under observation in the specific age period. We removed patients with no clinical data from this analysis.

We matched age-sex controls with 1:3 ratio from the neurology and the rheumatological departments. Mortality was higher in MG cohort compared to control groups (Rheumatology and neurology, *p* < 0.001, 31% vs. 4 and 7% deaths in each of the control groups, respectively) (Figure 1). Specifically, in the Neurology cohort, the median age at death was 76 years (range: 34–89); and in rheumatology, the median age of death was 87 years (range 49–97 years) (Table 1).

**Table 1:** Demographics in MG and control groups.

Variable	Myasthenia gravis (*n* = 436)	Neurology-general (*n* = 1,308)	Rheumatology (*n* = 1,308)	*p*-value
Age at onset^*^	55.7	57.97	56.33	Neuro:0.19, Rheu:0.18
Sex (F)	53% (231/436)	53.97% (706/1,308)	52.47% (686/1,308)	Neuro:0.7, Rheu:0.78
Death	31% (133/436)	7% (96/1,308)	4% (47/1,308)	*p* < 0.001
Death age median (range)	79 (32–107)	76 (34–89)	87 (49–97)	*p* < 0.001 for MG vs. rheumatology. *p* = 0.23 for MG vs. neurology group
The most common five diagnoses in the cohort	Myasthenia gravis	Ischemic stroke (344) TIA (106). Seizures and epileptic disorders (105). Intracranial hemorrhage (60). Migraine, cluster headache and other primary headaches (50)	Nonspecific arthritis/arthralgia (219) Rheumatoid Arthritis (80) Osteoarthritis (65) Scleroderma/systemic sclerosis (42) Dermato/polymyosits (42)	NA
Steroids	58.2% (254/436)	2.3% (30/1,308)	30% (392/1,308)	*p* < 0.05 between MG and Neuro, and between MG and Rheumatology
Non-steroidal immunosuppression (Azathioprine, Mycophenolate Mofetil, Methotrexate, Cyclophosphamide)	46.3% (202/436)	1.3% (17/1,308)	59.6% (779/1,308)	*p* < 0.05 between MG and Neuro, and between MG and Rheumatology
Chronic IVIG	17.2% (75/436)	0.15% (2/1,308)	1% (13/1,308)	*p* < 0.05 between MG and Neuro, and between MG and Rheumatology
Biological treatments (like Adalimumab, Infliximab, Rituximab, Eculizumab, and others)	9.6% (42/436)	0.22% (3/1,308)	21.9% (286/1,308)	*p* < 0.05 between MG and Neuro, and between MG and Rheumatology

The table presents the basic demographic features of the MG cohort and each of the disease control groups; neurological, and rheumatological patients. Each control group is composed of 1,308 patients (1:3 ratio with the MG cohort). The first two rows ensure the age and sex eligible matching between the groups, as these parameters were not statistically different. Besides age and sex data, information about incidence and median age of death were provided in the third and fourth rows, respectively. The table provides also information about the leading disorders within the control groups and the exposure to chronic medications like steroids, non-steroidal immunosuppressants and more. ^*^When diagnosis age was not available, the closest visit was used. In the control groups, this was the age of matching.

Mortality in the MG cohort was documented in detail for 48 out of 133 MG patients. In the rest 85/133 we could only know the date of death without other information; these 85 patients most probably died at other institutions or home. In 38% (18/48), death was MG-related. Notably, in only 4.2% (2/48) of patients MG crisis was the leading solitary cause of death. In 33.3% (16/48), additional severe acute illnesses were found in addition to MG exacerbation, including sepsis and septic shock, severe pneumonia, pleural effusion, pulmonary embolism, chronic obstructive pulmonary disease (COPD) exacerbation, acute renal failure, and tension pneumothorax. Overall, the leading systemic cause of death was infectious or sepsis in 52% (25/48), followed by end-stage oncologic status in 23% (11/48) and cardiovascular illness in 15% (7/48), *p* < 0.01 for infectious etiology vs. all the others. Out of the 52% (25/48) patients that died due to infectious etiologies, a remarkable 92% of them were chronically treated with steroids, compared to 58% (254/436) in the overall cohort (*p*-value < 0.01). No significant changes were found for all the other maintenance medications.

### Mortality associations in MG

**Figure 2:**
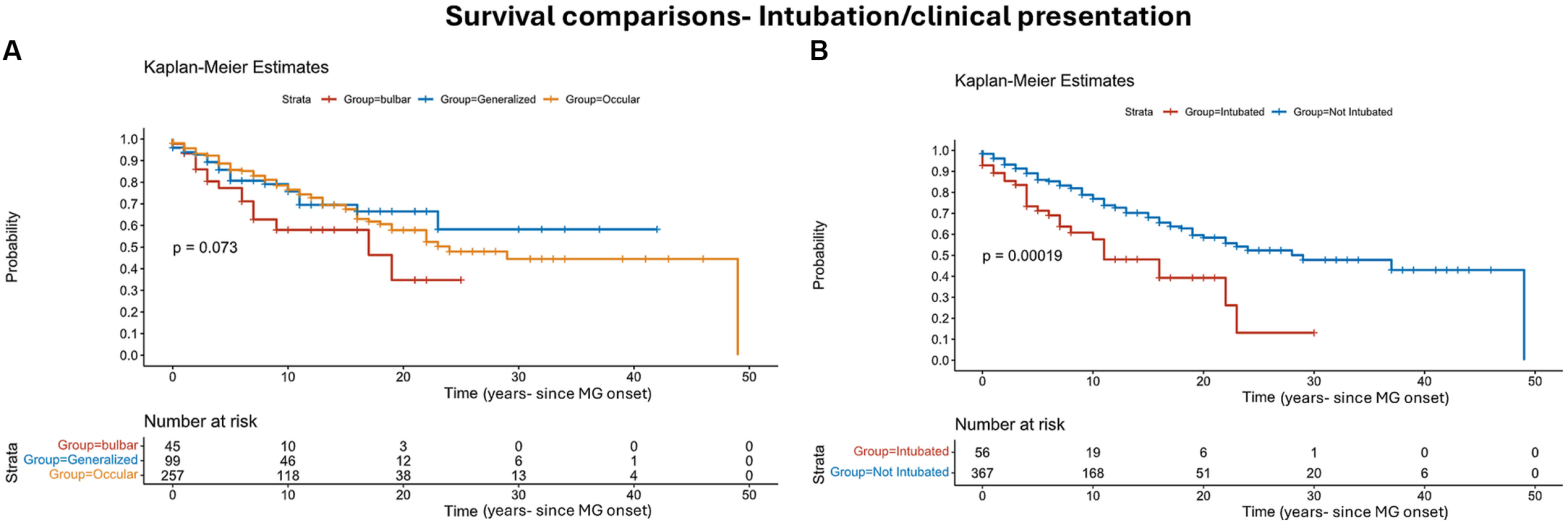
Survival risks comparison: Kaplan–Meier survival analysis comparing the overall survival between patients with MG symptoms onset, ocular/strictly bulbar/generalized **(A)**, and patients who were intubated due to myasthenic crisis **(B)**. The informative risk table set (bottom) displays the number of patients that were under observation in the specific age period. Note that the time axis in this figure refers to ‘time from MG onset’.

Serological status, electrophysiological features, intubation during crisis, and whether patients had thymoma/ thymectomy, are parameters that were investigated for association with mortality in MG patients. The cohort was divided, according to clinical presentation, into ocular, generalized, and bulbar (257, 99, and 45, respectively). Mortality at 5 years from onset was 20% (9/45) at the bulbar MG vs. 12.84% (33/257) for the ocular MG, and 17.17% (17/99) for the generalized MG. Bulbar onset MG showed the highest mortality in general (Figure 2A), however not reaching statistical significance at 5, 10, and 15 years (*p* = 0.12, 0.08, and 0.16, respectively, Table 2).

**Table 2:** Mortality associations in MG.

Variable	Serology		Presenting symptom			Intubations due to MG crisis		Thymectomy/thymoma		
	Pos (AChR)	Double-Neg	Ocular	Generalized	Bulbar	Yes	No	No thymectomy/thymoma	Thymectomy without thymoma	Thymectomy with thymoma
Mortality 5 years of onset	8.3% (14/168)	6.3% (3/48)	12.84% (33/257)	17.17% (17/99)	20% (9/45)	26.8% (15/56)	12.5% (46/367)	19.2% (55/286)	3% (3/100)	7.5% (3/40)
Mortality 10 years of onset	11.38% (19/168)	12.5% (6/48)	19.06% (49/257)	20.2% (20/99)	28.9% (13/45)	35.7% (20/56)	18.3% (67/367)	28% (80/286)	4% (4/100)	7.5% (3/40)
Mortality 15 years of onset	14.37% (24/168)	14.58% (7/48)	23.34% (60/257)	23.23% (23/99)	31.11% (14/45)	41.1% (23/56)	22.6% (83/367)	31.8% (91/286)	7% (7/100)	20% (8/40)
*p* value	*p* > 0.5 for all		*p* = 0.12 for 5-years mortality. *p* = 0.08 for 10-years mortality *p* = 0.16 for 15-years mortality (all between ocular and bulbar).			*p* = 0.03 for 5-, 10-, and 15-years mortality		*p* < 0.01 for all		

Variable parameters were analyzed and their association with mortality in the MG cohort. These parameters include (from left to right columns) serological status, clinical presentation, intubation due to MG crisis, and thymectomy addressing also thymic pathology results.

Serological subtypes were not associated with mortality. MUSK seropositive patients were excluded from this analysis due to their small number (only six patients). Analyzing according to time from symptoms onset showed 8%, 11%, and 14% mortality after 5, 10, and 15 years, respectively, in the seropositive group (168/222) and 6, 13 and 15% in the double-seronegative group (48/222) (Table 2). Jitter analysis showed that mortality at all the time intervals was lower in patients with average jitter below 20 μs, *p* < 0.05, otherwise, no significant differences were found between the other subgroups of averaged jitter (20–29 μs, 30–39 μs, ≥ 40 μs).

Intubation due to MG crisis was performed in 13% (56/422). We excluded elective intubations or acute intubations not related to MG crisis. Mortality was higher in the intubated group and was as high as 46% (26/56) compared to non-intubated group 27% (100/367), *p* = 0.0001 (Figure 2B). Mortality at 5 years from onset was 26.8% (15/56) in the intubated group vs. 12.5% (46/366). This difference also holds true at the 10 and 15 years from onset: 36 and 41% at the intubated group vs. 18% and 23% at the non-intubated group, *p* = 0.06, 0.03, 0.04 for the 5-, 10-, and 15-years mortality, respectively (Table 2).

**Figure 3:**
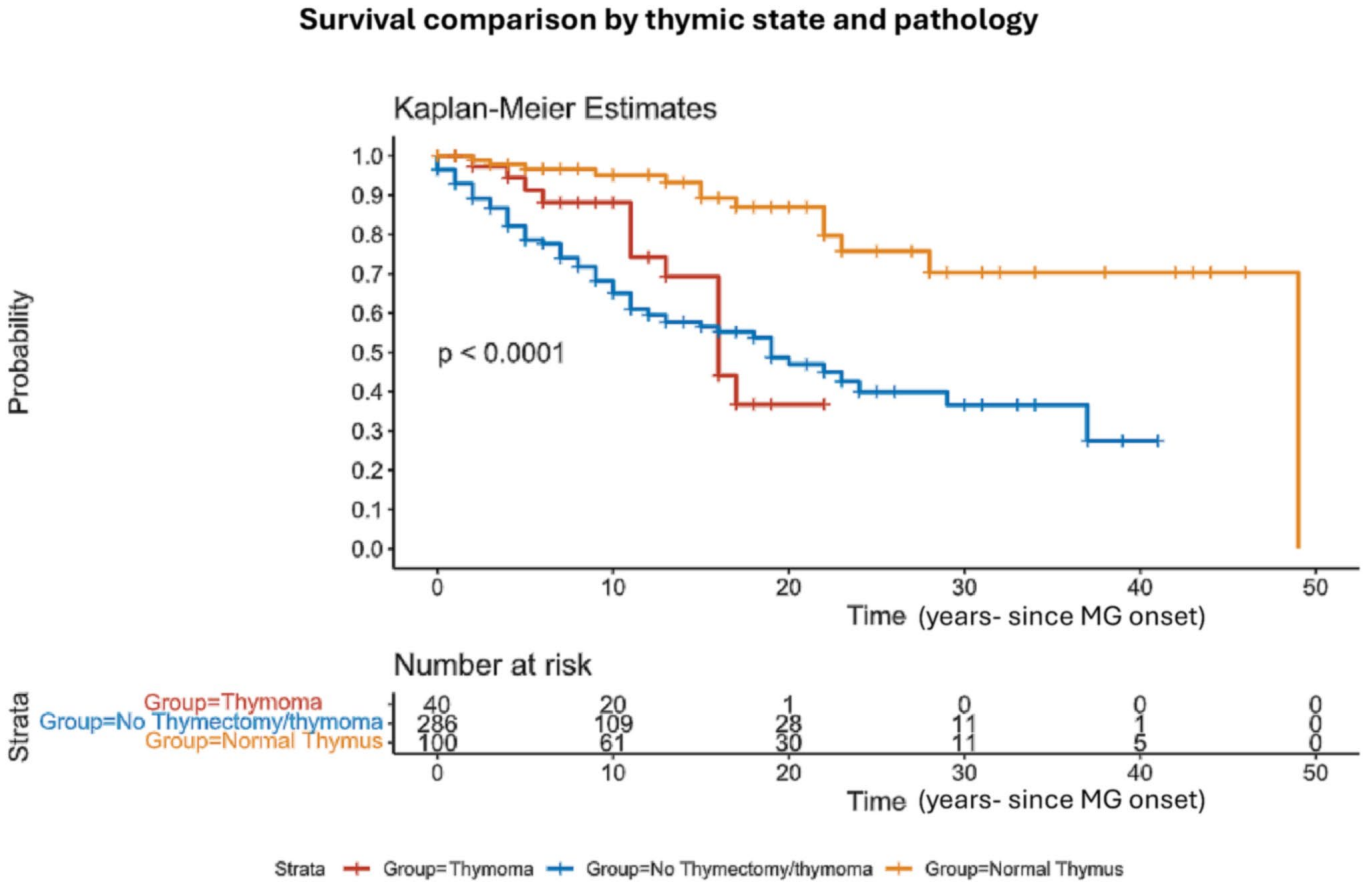
Survival comparison by thymic state and pathology: Kaplan–Meier survival analysis comparing the overall survival between patients who underwent thymectomy and patients with no thymectomy. The informative risk table set (bottom) displays the number of patients that were under observation in the specific age period. Note that the time axis in this figure refers to ‘time from MG onset’.

We have also classified patients by thymic removal vs. no thymic removal, with 32% of patients undergoing thymectomy. Mortality was significantly higher in patients who preserved their thymus 35% (101/286) vs. 19% (26/140) *p* = 0.0001 in the thymectomy cohort. We further divided the thymectomy group into two subgroups of thymectomy with and without thymoma (based on pathological and oncological reports) (Figure 3). The three subgroups regarding thymus status included patients without thymoma/thymectomy (286/422), thymectomy without thymoma (100/422) and thymectomy with thymoma (40/422). At all the investigated time intervals, mortality was higher in the first group (19%, 28%, and 32% for the 5, 10, and 15 years, respectively). At the thymectomy without thymoma the ratio was 3%, 4%, and 7%, and for the third group, the mortality was 8%, 8%, and 20%, *p* < 0.001 (Table 2).

Comparing mortality rates among patients with symptom onset before 2010, between 2010 and 2020, and after 2020 showed no significant differences. We conducted this comparison to investigate whether there were any dramatic negative changes in MG mortality due to the COVID pandemic, or possibly positive changes due to the accumulated experience in managing MG patients and the biological treatments emerging for MG that became available in the past few years. These treatments include Rituximab (39 patients) and Eculizumab (three patients). More novel treatments, like Ravulizumab and Efgartigimod, became available only at the edge of the research-defined time or immediately after, and thus were not counted. The mortality rates within 3 years were 9.8% (6/61), 11.6% (22/189), and 6.3% (11/175) for patients with onset after 2020, between 2010 and 2020, and before 2010, respectively, with a *p*-value greater than 0.05. This lack of statistical difference persisted when examining mortality within 5, 10, and 13 years, comparing onset before 2010 to onset between 2010–2020 (with the group onset after 2020 being irrelevant for these comparisons).

## Discussion

In this single-center database-based study, we compared mortality among 436 patients with MG diagnosed between 2000 and 2023. Overall mortality rate was higher in MG patients compared to age-sex-matched disease controls of rheumatology and neurology cohorts (Figure 1). The novelty of our study lies in the inclusion of an age and sex-matched disease control group originating from similar demographic area and receiving similar medical care and screening programs, which in turn improves causality assessment and bias mitigation in determining whether the MG is a causal factor contributing to increased mortality.

To date, a limited amount of research has looked at crude mortality rates in MG, with a majority of studies not implementing control groups at all or only comparing to the general/healthy population, which can be misleading. Using matched control groups is crucial for the mitigation of the immunological effect of chronic disease indirectly related to MG (achieved via the neuro group). In addition, matched control groups can be exploited to alleviate the effect of chronic direct autoimmune systemic conditions and the use of immunosuppressants (achieved via the rheumatologic group). Few large registry studies based on death registries (20, 21) and one based on insurance records, estimating mortality in MG as high as 8 per million in 2018 (22). Other studies used large national surveillance methods to report 2 deaths per million in 2023 (8). With limited clinical information, these studies might have inaccurately under or over-diagnosed. A large population-based study reported median age of death of 16 years younger compared with the local general population (59.5 and 75.47 years, respectively, *p* < 0.05) with older MG patients having a higher mortality rate (9). Compared to this study, the median age of death was older in our cohort (79 years). We believe this difference relates to differences in medical infrastructure and the availability of new recent biological treatments in refractory MG patients.

Despite having a general increase in mortality, time to death was not the shortest in MG group; median age of death in MG was 79 years vs. 76-neurological, and 87 rheumatological patients. This means that patients in the MG groups died more, in general, than patients in each of the control groups. However, when patients die in the general neurological group, they die younger than MG patients. Severely acute diseases, causing dramatic deterioration and death, including acute ischemic or hemorrhagic major strokes, epileptic status, myocardial infarction, multiorgan failures in patients at the intensive neuro unit, and more, can explain the younger age of death in this control group.

Mortality was also time-related. In our cohort, 5 years from MG onset, mortality was as high as 14%, increasing to 21% 10 years from onset. This is higher than reported in a study of over 1,000 MG patients in which the overall mortality rate during the first 5 years was 2.4 and 5% at 10 years (9). A possible explanation of this discrepancy is the bias to more severe or refractory MG patients referred to our tertiary center, while mild cases continued follow-up at smaller centers or even at general neurological clinics. The higher mortality in the first few years of the disease might be related to unstable disease with a higher incidence of myasthenic crisis (4). There were no significant sex differences in survival, with similar rates between men and females, conflicting with other studies showing higher mortality rates for men (5), explained by disease affecting older men and younger women. On the other hand, other studies showed higher mortality in females according to the ‘Relative Standardized Mortality Ratio’/SMR that took into account the expected number of deaths according to age (23).

Whether clinical, serological, or electrophysiological parameters are associated with mortality in MG is not fully understood. These associations may have a crucial clinical impact if considered as risk factors for mortality in MG patients. Clinicians will have to consider more effective treatments if an MG patient presents one or more of these associations. Our data sheds light on the association of intubation due to respiratory failure in MG crisis as a possible risk factor/association, similar to what was reported by Alshekhlee et al. (4) for in-hospital mortality in MG. To our knowledge, no previous study suggested this association in MG in general. An additional positive association we found was the lack of thymectomy. These results are not consistent with the recently published results of Kooshesh et al. (12). This discrepancy may be due to several factors, including different populations of patients (they had mostly cardiac patients instead of MG patients). Regarding clinical presentation of MG, bulbar onset MG showed higher mortality but did not reach statistical significance (Figure 2A). Serological status was not associated with mortality in our study, challenging previous findings that claimed such an association with an even titer-dependent manner of the AChR (24). The average jitter did not show a prominent correlation with mortality. We only found that in the minority of MG patients, in which the averaged jitter was below 20 μs, mortality was inferior to other subgroups of jitter without differences between the other subgroups (20–29 μs, 30–39 μs, ≥ 40 μs). A possible explanation for this result is that patients with an average jitter of below 20 μs are very mild or controlled cases or cases in remission, that are less prone to exacerbations. Interestingly, mortality was not changed between 2020 and 2023 compared to before. This means that it was not heavily affected by the COVID pandemic but neither improved with the novel biological treatments. The latter statement might be too early to deduce after only 3–4 years as more time and experience need to be accumulated to properly assess the effects on mortality under the novel biological treatments.

Immunosuppressant therapies are commonly used to manage MG, yet their impact on mortality risk remains uncertain. Theoretically, chronic immunosuppression is correlated with pro-oncogenic and pro-infectious states that may burden survival. We aimed to normalize this factor by implementing the age-sex matched control group; patients addressed to the rheumatologic department in our institution. This group is composed of patients with variable systemic autoimmune disorders receiving chronic immunosuppressants. Patients in this group received, on average, more non-steroidal immunosuppressants and biological treatments, than MG patients (Table 1). Comparing the MG cohort with the rheumatological group shows higher mortality in the MG cohort (Figure 1). If this result is due to the differences in the ratio of chronic immunosuppression, we would expect it to be the opposite, thus, this issue is probably not strongly linked to the difference between the groups regarding mortality. Nevertheless, data in this regard remains sparse, necessitating future research to delve into the nuances of this relationship.

Our current study has several limitations. First, this is a retrospective case control study which might have reported bias due to their reliance on past noted patient self-reported data. Second, the single tertiary center fashion of the cohort possibly resulted in a bias toward more difficult cases, while mild cases continued their follow-up at smaller centers or regular neurological clinics. Furthermore, we observed relatively low rates of overall mortality for rheumatological patients which we assume can vary widely depending on the specific rheumatic condition. Additional limitation relates to the lack of monitoring of phenotypic change or conversion with time. Some patients who initially present as ocular MG may convert to a generalized phenotype over time. This might have affected the reliability of our secondary outcome regarding mortality associations in MG with clinical presentation/phenotype. However, this issue has no expected effect on the primary study outcome, which is the overall mortality in MG compared to the matched control groups. On the other hand, our study strength is using two matched different disease controls to optimize epidemiological outliers that might affect mortality rates such as medical disparities and access to medical facilities as well as the long follow-up with detailed medical history.

In conclusion, our study suggests, by using reliable disease age- and sex-matched control groups, higher mortality in MG patients compared to disease controls, especially in the first 5 years of the disease. Associations between mortality in MG and other parameters were established, including clinical presentation of bulbar weakness, thymic removal, and more. Our data also showed that mortality has not significantly changed since the beginning of the century, despite treatment progress, and the COVID pandemic. Further research is needed to clarify this relationship between MG and mortality and its associations.

## Data Availability

The raw data supporting the conclusions of this article will be made available by the authors, without undue reservation for authorized investigators.
